# Latent Occupational Burnout Profiles of Working Women

**DOI:** 10.3390/ijerph19116525

**Published:** 2022-05-27

**Authors:** Maciej Załuski, Marta Makara-Studzińska

**Affiliations:** Division of Health Psychology, Faculty of Health Sciences, Collegium Medicum Jagiellonian University, 31-008 Kraków, Poland; marta.makara-studzinska@uj.edu.pl

**Keywords:** woman, occupational burnout, latent profile analysis, emergency call-taker and dispatcher, Person-Oriented approach, seniority, marital status, fertility

## Abstract

According to scientific research, emergency call-takers and dispatchers (ECD) are particularly vulnerable to burnout syndrome. It can be observed that this occupation is predominantly performed by women. Moreover, the studies on occupational burnout indicate its different causes depending on employees’ gender. The aim of this research was to apply a Person-Oriented approach in order to examine the relationships between particular risk factors, the level of burnout, and its health consequences in a group of women. A cross-sectional survey study was conducted on 296 women (call-takers and dispatchers) from public-safety answering points in Poland. The Link Burnout Questionnaire and a sociodemographic questionnaire were used to gather information. The method of latent profile analysis (LPA) was employed in the study. The study revealed burnout patterns without full symptoms as well as four different burnout profiles. The findings partially confirmed initial assumptions about correlations between the length of service as ECD, marital status, motherhood, burnout symptoms, and body mass index (BMI). Sociodemographic variables differentiated the examined women in terms of their emotional exhaustion and BMI. Three groups of women at risk of burnout and overweight were identified: those with the shortest job experience, those with the longest job experience, and an intermediate group. In each of these groups, symptoms indicating a possible risk of burnout-related health issues could be observed. The application of a Person-Oriented approach allowed for assessing possible correlations between burnout risk factors, its symptoms, and health consequences.

## 1. Introduction

Employment rates in the group of Polish women aged between 25 and 49 is currently similar to the rate in other EU countries [1]. However, economic activity refers mainly to women with a higher education who have no children or only one child. Since 2016, the female employment rate in Poland has been declining both in absolute terms and in comparison to EU average standards [1]. The most common reasons explaining such a tendency are difficulties in combining work and caring responsibilities, inflexible working hours, expectations of fulfilling social roles and unhealthy working conditions, which increase occupational stress [2]. Working women report more tension experienced in the workplace than men do; they feel more exhausted in everyday life, (vital exhaustion), more depressed, and burned out [3,4]. A meta-analysis of the study results revealed that gender determines the prevalence of selected symptoms of occupational burnout. Women are more likely than men to report symptoms of physical and emotional exhaustion from work [5]. It is believed that women are more prone to stress reactions, although not all researches support this assumption. The differences may relate to different patterns of burnout rather than global outcomes. Women report higher levels of emotional exhaustion, whereas men are more likely to report depersonalization in work relationships [6]. This fact is explained by a greater expression of emotional tension in women and men’s secretiveness. The suppression of emotional tension in men is revealed in their depersonalization of other people in professional relationships [5].

Trying to identify burnout risk factors in women, typical demographic characteristics such as marital and parental status were examined. However, their role as direct burnout predictors was not confirmed [7]. While looking for indirect relationships, the importance of social expectations towards women and their partners was examined as well. Fulfilling the traditional role of a housewife along with a professional job seemed to increase the risk of burnout. However, if a woman perceived her work and family life as equivalent social roles, the risk of burnout had a tendency to decrease. This was confirmed by the results of a study on women employed in different service sectors. The mediating variable of work-home conflict (WHC) was shown to influence both the level of burnout and the incidence of family problems [8]. The level of occupational stress in the female group was found to depend less on the respondents’ gender and more on the complexity of the roles performed both at home and at work [9]. Moreover, significantly fewer (26%) working men declared helping with household duties when compared to 63% of working women [10]. Similar differences recurred in the study of childcare declarations (19% of men and 66% of women).

The issue that is not sufficiently explained is the reasons behind women’s occupational burnout. They may include the incidence of the aforementioned WHC conflict or unsatisfied professional expectations. Chesak et al. [11] found that women experience occupational stress and burnout for different reasons than men do. The source of burnout for working women may be trying to meet cultural expectations of non-work-related roles, but also prejudice and barriers blocking their free professional development. Female workplaces are characterized by higher monotony, lower participation in planning, higher demands, as well as stronger exposure to social criticism, higher risk of psychological and sexual harassment and, at the same time, lower earnings, lower prospects for career advancement, and greater job insecurity [12,13]. The confirmation of a latent relationship is provided by the results of a study conducted in a group of women employed in health care. They showed that emotional exhaustion and depersonalization mediate between work-related pressure (WHC) and psychological disorders in working women [14]. Therefore, taking into account gender and social roles in the search for burnout predictors seems to be a vital research direction.

### 1.1. Body Mass Index

Relatively little research has been devoted to the relationship linking occupational burnout to employees’, particularly women’s, weight and eating behaviors. The results obtained have not always confirmed the researchers’ assumptions. It was found out in one of the studies that higher levels of occupational burnout co-occurred with an increase in so-called emotional eating and uncontrolled eating, which can lead to obesity [15]. A study of a large group of working women showed that obese female employees were more emotionally exhausted and, as a result, were more likely to be on sick leave [16]. Emotional exhaustion, therefore, played a mediating role in the relationship: high BMI-frequent sick leave. However, in a longitudinal study of a group of women with no health problems, the results contradicted the hypothesis that obesity predicts burnout and burnout is a predictor of obesity. Weight loss, which was found to accompany burnout, was explained by hypocortisolism, which is typical of burnout and leads to limitations in food intake. This hypocortisolism may be present in the patients with more severe burnout symptoms [17]. However, it is known that occupational stress often leads to the incidence of a metabolic syndrome, including the most significant condition, which is abdominal obesity. This fact has been confirmed by several scientific studies [18]. Smoking habit analysis revealed no significant differences in burnout levels, but alcohol consumption above health limits significantly increases the level of emotional exhaustion. [19]. It should also be added that the findings of studies conducted on emergency workers of both genders (firefighters, police officers, 911 operators) frequently indicate that symptoms of overweightness and obesity are common in these occupational groups [20].

### 1.2. Age and the Length of Service as ECD

Correlations between occupational burnout and respondents’ age and the length of service as an ECD take linear and non-linear forms for various burnout symptoms [21]. A study of a large sample of Canadian workers of both genders revealed that the severity of depersonalization (cynicism) symptoms and professional inefficacy increased with the age of the subject and their length of service. In the case of women, the relationship between age and emotional exhaustion was bimodal. Female employees aged between 20 and 35 and those over 55 revealed higher levels of emotional exhaustion than the others. As this relationship did not apply to men, the results were explained by the specificity of women’s life stages. The level of education, marital status, the number of children and the number of working hours tend to change with age. A study conducted in a large group of working Swedish women aged 18 to 64 years showed the highest prevalence of burnout symptoms in the youngest age group: 20–34, and the lowest in the 50–64 group [12]. Research indicates that higher levels of burnout among women may be observed in those who are younger, have less work experience, lower education, are not in a stable marriage or partnership, and for those whose health is poorer; that is, in women with lower financial, emotional, and somatic health resources [12,13].

The emergency call-taker and despatcher (ECD) profession is one of the most stressful jobs [22,23]. The reasons for this include contact with the suffering of another human being, which requires immediate and responsible decisions in unclear situations, lack of feedback on the effectiveness of the actions taken, the need to maintain emotional neutrality while being exposed to verbal aggression, and traumatic behavior of the callers. Adverse working conditions such as shift work, sedentary work, insufficient recovery breaks between emotionally taxing conversations, and time pressure make it difficult to reduce occupational stress and contribute to a number of health issues including mental disorders and obesity as a result of a metabolic syndrome [20,24]. The ECD profession requires developing individual strategies of coping with stress, however, due to organizational deficiencies, newly hired employees are not sufficiently prepared for work and do not have sufficient training dedicated to mental health protection. A critical factor is also the lack of support from employees’ superiors including the lack of feedback on the results of actions taken [25]. Characteristic features of this profession include the predominance of women, the age range of 25–45 and employees with secondary or higher education [26,27]. How do women cope with such an emotional burden in this profession?

In the research on burnout, it is multivariate models that are primarily used. They are complemented by the so-called Person-Oriented approach, which is opposed to the Variable-Oriented approach [28]. The Person-Oriented approach allows the researcher to focus their attention on the differences in the results obtained rather than on the similarities between the data and a predetermined pattern. The pattern may be the results exceeding the cut-off point for burnout or no burnout diagnosis. An analysis of the results excluding their conformance to the pattern makes it possible to distinguish classes of subjects who are characterized by different intensity of the results obtained in each dimension. The distribution of the results, in particular burnout dimensions, in each class creates a specific burnout profile. The diagnosis of burnout does not necessarily imply the presence of identical and equally severe symptoms in all individuals due to individualized etiology, and because of the heterogeneity of the subjects in terms of their average level of burnout [29,30,31]. The aforementioned approach allows for comparing latent burnout profiles (without all symptoms) within one professional group, testing assumptions about the chronology of the incidence of burnout symptoms and trajectories of changes [32], and for identifying typical and atypical burnout patterns (inconsistent patterns). It also makes it possible to describe groups which do not fit the classical approach to burnout categorization (according to the rule: low, medium, high) [32]. It provides an opportunity to learn about individualized ways of experiencing work environment conditions.

A variety of statistical methods is employed in profile analysis ranging from cluster analysis and logistic regression analysis to latent class analysis (LCA) and latent profile analysis (LPA). LPA consists of dividing observations into mutually exclusive classes using the distribution of the scores obtained for different variables and estimating their fit parameters [33]. It allows for an analysis of both qualitative and quantitative data and the most accurate identification of the number of classes, thus ensuring that their size is adequate [32]. Although initially the Person-Oriented approach was used to analyze only the dimensions of burnout, LPA allows us to study more variables, including qualitative ones. The variables which are examined include work engagement, depressive symptoms, or coping strategies as well as employee personality traits [31,32,34,35,36].

Our study was aimed at employing a profile approach to determine the cause–burnout–health-consequence relationship. We hypothesized that the causes of burnout among women include those related to balancing caregiving and professional roles, while the consequences include changes in eating habits which may lead to overweightness. The presence of WHC conflict was operationalized in terms of two variables: marital status and the number of children under respondents’ care. The disease consequences were operationalized in terms of body mass index (BMI). The inclusion of the variable of the length of service was aimed at capturing changes in different periods of time. The study controlled the effect of variables such as age, the number of working hours per week, and education. However, these variables were not included in the statistical analysis. In this study we decided to answer the following research questions:

1. Can different classes and profiles be distinguished in the group of female call-takers and dispatchers based on their burnout symptoms, BMI, the length of service, marital status, and the number of children under respondents’ care?
2. Do the length of service as ECD, marital status, and the number of children allow for predicting differences between profiles?
3. Do differences between profiles allow for predicting the incidence of health disorders such as overweightness and obesity?

## 2. Materials and Methods

### 2.1. Subject

The examined group consisted of 315 female ECD workers employed in 14 public safety points (PSAP) in Poland. After removing incomplete data, 296 result sets were included in the analysis. The average age of surveyed women was: 34.37 ± 7.73 years. The age ranged from 19 to 65, whereas 35.5% of the women belonged to the age range below 31 years, 42.59% to the range of 32–40 years and 14.92% to the range of 41–45 years. A proportion of 7.3% of the respondents were over 46 years old. As far as respondents’ education is concerned, 78.04% of ECDs had bachelor’s and master’s degrees, 21.62% had secondary education, and 0.34% had vocational education. The average number of weekly working hours in the study group was 42.35 ± 6.22, in the range of 12–60 working hours. Further information about the study group is included in the Results section (Table 1).

### 2.2. Applied Research Tools

The Polish version of Massimo Santinello’s Link Burnout Questionnaire (LBQ) adapted by the Psychological Test Laboratory of the Polish Psychological Association [37] was employed to assess the level of burnout. The tool consists of 24 statements to which the respondent refers using a 6-point Likert-type scale (1—never, 2—rarely, 3—once (or more) per month, 4—about once a week, 5—several times a week, 6—every day). The questionnaire contains 4 subscales corresponding to 4 dimensions of professional burnout. They include the following dimensions: psychophysical exhaustion (PE), relations deterioration (RD), professional ineffectiveness (PI), and disappointment with work (DI). PE is a dimension which refers to employees’ psychophysical resources. One end of this scale describes a state of exhaustion, fatigue, and a feeling of being under pressure to perform, while the other end describes a state of activity and energy. RD refers to the quality of interpersonal relationships with service recipients. One end describes the subjective treatment of callers, indifference, distance, and even hostility, whereas the other end expresses a commitment to relationships and an individual treatment towards each caller. PI is a dimension referring to self-assessment of one’s professional competence. One end of the dimension is characterized by a sense of ineffectiveness and lack of work results, therefore a sense of a lack of professional effectiveness, whereas the opposite end is characterized by effectiveness in achieving professional goals. DI is a dimension of existential expectations related to the employee’s motivation behind choosing a caring profession aimed at helping other people. There are individuals who perceive helping as a mission and see themselves as the ones who do good. When confronted with the reality of the workplace (uncooperative callers, inability to help everyone), they can experience disillusionment. One end of the dimension describes disillusionment and a lack of enthusiasm, whereas the opposite end describes a state of passion, enthusiasm, and job satisfaction. The theoretical range of each subscale is between 6 points (score indicating low levels of a negative tendency) and 36 points (maximum intensity of a negative tendency). The LBQ provides 5 indicators: the higher the score on each subscale, the greater the intensity of a negative tendency on each of the 4 dimensions of burnout. An additional fifth indicator of burnout is the total burnout score, the so-called burnout syndrome composite index (LBQINDEX). It is the sum of the scores obtained in the 4 subscales of the questionnaire. This index was not used in the study. Cronbach’s α reliability coefficient ranged from PI = 0.66, RD = 0.67, PE = 0.80 to DI = 0.85.

Sociodemographic data were obtained using a separate questionnaire. They included: respondents’ age, education, length of service as an ECD, weekly number of working hours, marital status, and the number of children they had. In the further analysis the data from married women and those living in a partnership were examined together. The other group consisted of single, widowed, and divorced women. Women with children were combined into one group regardless of the number of children. However, this grouping was carried out after the LPA analysis, as a supplementary one. Women’s age, education, and their weekly number of working hours were control variables not included in further analyses.

### 2.3. Ethics

The study protocol was approved by the Bioethics Commission at Jagiellonian University Medical College (decision No. 1072.6120.23.2017) and was carried out in accordance with the recommendations of the APA Ethics Code.

### 2.4. Procedures

The data for this study were collected between January and May 2020 among emergency call operators working in 17 PSAPs in Poland. In total, 800 sets of research tools were mailed to their workplace. Participation in the study was anonymous and voluntary. Five hundred fifty eight sets of questionnaires were returned, of which 546 were correctly completed. Of this sample, 315 were from women. After removing incomplete data, 296 result sets were included in the analysis. Questionnaires were collected from 14 PSAPs in Poland. Only the ones completed by women were selected for further analyses.

### 2.5. Statistical Analysis

The LPA method was used to divide female subjects into comparable classes by entering all predictors together. The assignment of particular observation methods to identified classes was based on the mean values of the 4 dimensions of burnout as well as BMI, the length of service as ECD, marital status, and the number of children living at home.

The model fit was estimated employing the following indices: the Akaike Information criterion (AIC), the sample-size-adjusted Bayesian Information Criterion (BICa), and the bootstrapped Lo-Mendell-Rubin test (BLMRTp) [38]. More accurate models are characterized by low AIC and BIC values and higher Latent class probability [range] and Class prevalence [range] values. The BLRT(p) value compares a model with a given number of classes to a model which is one class lower. A significant *p* (*p* < 0.05) means that a model with a given number of classes is a better fit than a model which is one class lower. We also evaluated the value of entropy index, assessing how accurate a model is at classifying people into latent profiles (the higher value within the range 0–1 indicates better accuracy). The analysis presented above was performed twice on unconstrained models and on constrained models. An unconstrained model means that parameters of the model can take any values, and are not imposed by the researcher in advance. In constrained models it is assumed that each variable affects class membership to the same extent.

The Chi-squared test was used to compare qualitative variables among groups. In case of low values in contingency tables, the Fisher’s exact test was used instead; the Kruskal–Wallis test (followed by Dunn post-hoc test) was used to compare quantitative variables between more than two groups. The significance level for all statistical tests was set at 0.05. The statistical software R 4.1.3. [39] with tidyLPA package was used for computations [40].

## 3. Results

### 3.1. Characteristics of the Group by the Variables Used in the Analysis

Table 1 presents the data characterizing the group in terms of the variables included in the analysis. Respondents’ length of service as an ECD ranged from 1 month to 12 years. Most of the women had work experience of 5 to 8 years (35.5%), followed by those with the length of service between 1 month and 3 years (27.30%), 3 to 5 years (27%), and finally over 8 years (10.20%) The group was dominated by women living in domestic partnerships (69.53%) and childless women (60.64%). The results obtained in the study group in the four dimensions of burnout were at the average level: PI-5 stanine, DI-6 stanine, and higher level: PE-7 stanine, RD-7 stanine. Regarding the BMI values, 3.8% of women were underweight (BMI < 18.5), 60.9% had normal weight, 31.8% were overweight, and 7.3% were obese (BMI > 30.0).

### 3.2. Profiles of Burnout

In the analysis, a number of nested LPA models were tested to select the best fit to the data. Starting with a model with one class, the nested LPA models were tested by adding one class at a time, first in the unconstrained group and then in the constrained group.

Unconstrained models were adopted for further analysis due to better AIC and BIC, more favorable entropy values, and latent class probabilities. Constrained models generated less numerous groups (class prevalence [range] values), below the assumed value of 5% of observations. These models assume identical effects of the variables analyzed on class membership, suggesting that all variables measure the same thing. In reality, the variables differ between one another (Table 2). The best fit was obtained by the unconstrained model with four classes, ranging in size from 24 to 161 observations (Table 3).

Due to a large number of variables and the use of both quantitative and qualitative variables, it was decided to abandon the graphical presentation of the results and use a tabular presentation. Table 3 shows the characteristics of the classes according to the variables used in the study and the significance of differences between the variables in each class.

The classes were significantly different as far as the following variables were concerned: BMI, the length of service as ECD, PE, RD, and DE, while they did not differ in terms of variables such as PI, marital status, and the number of children at home (Table 3). The following characteristics of the classes was conducted using the length of service as ECD as a starting point. Class 1 was formed by women with the shortest work experience (5 months on average) of normal weight, the most disillusioned with their job, with increased rates of emotional exhaustion and deterioration of work relationships. Job disappointment and job dissatisfaction reached high levels in these women. The class was named: unsuited, at risk of changing careers. Class 3 consisted of women with the length of service of about 3 years, of normal weight, the most emotionally exhausted, with signs of the greatest deterioration of professional relations, but still with a sense of professional mission. Psychophysical exhaustion and distancing when interacting with callers reached a high level in these women. The class was called emotionally exhausted and distanced but with a sense of mission. It was the least numerous group. Class 4 was formed by women with work experience of about 4 years, of normal body mass index, with average indicators of emotional exhaustion, the lowest index of deterioration of professional relations (also average results), and average disappointment with their work. The class was called: adjusted, with lack of enthusiasm. Class 2 consisted of women with the longest work experience (5 years on average), overweight, with an average rate of exhaustion and deterioration of work relations. The class was named: at risk of obesity and exhaustion. This was the most numerous group. What is particularly noteworthy is the low level of professional inefficacy and expected job outcomes observed in all women surveyed regardless of class affiliation. Marital status and the number of children at home did not differ much between particular classes. However, single women were definitely dominating in the class with the shortest length of service named: unsuited, at risk of changing careers (Class 1) and the class with 4 years of service named: adjusted, with lack of enthusiasm (Class 4). In the other two classes, the ratio of coupled women to single ones was roughly similar. As far as the number of children is concerned it has already been mentioned that childless women dominated the whole examined group (60.64%). The highest number of childless women was observed in the class with the shortest time of service (Class 1) and the Class with 4 years of service named: adjusted, with lack of enthusiasm (Class 4). At the same time, these were the classes in which unmarried women predominated. The highest number of women raising children was in Class 2, with the longest length of service, overweightness, and at risk of exhaustion. Women living in relationships reported significantly lower BMI (Chi-squared test = 6.22, df = 1, *p* = 0.016), when compared to single women. After separating childless women and women with children (from Classes 1 to 5) into two groups, it turned out that motherhood statistically significantly co-occurred with higher PE (Chi-squared test = 4.09, df = 1, *p* = 0.042) and higher BMI (Chi-squared test = 6.341, df = 1, *p* = 0.011).

## 4. Discussion

The results of the conducted study revealed the risk of some problems related to occupational burnout in the whole group of female call-takers and dispatchers. The risk was related to the depletion of psychophysical resources, the inability to replenish them and, consequently, the risk of losing energy to work. In the examined group there were also signals indicating the risk of depersonalization towards callers and treating them with distance or indifference. In some of the respondents, symptoms of already developed psychophysical exhaustion and the presence of distancing and cynicism in relations with callers were recognized. Some of the women were disillusioned with their jobs and reported a lack of job satisfaction as a consequence of a mismatch with their values and life ideals. As observed by Artz et al. [7], lower levels of burnout may apply to so-called modern women who treat work as a significant part of their lives. It is different in the case of women for whom professional work does not conform to the traditional role model of a woman trying to balance domestic responsibilities with the demands of professional work. In our study, the representation of the group of modern women can be found among unmarried, childless respondents, most of whom belonged to Class 4. However, this is only a guess, and is not confirmed by significant results of the research. This is especially pertinent, as in this class there was the largest number of women having two children.

According to the assumptions of Hypothesis 1, it was possible to differentiate the studied group and divide it into classes by means of variables whose influence had been presumed. The classes which were formed this way were characterized by variable values that formed logical-looking profiles. However, not all variables allowed for class differentiation. The assumption (Hypothesis 2) about a relationship between the length of service as ECD and different symptoms of burnout was confirmed. Taking into account BMI, marital status, and the number of children, the highest levels of emotional exhaustion were identified within two ranges of the length of service as ECD: up to 3 years and the shortest one (5 months on average). Longer service as ECD (up to 3 years) co-occurred with a higher intensity of aforementioned symptoms. The length of service of up to 3 years was also found to co-occur with more intense symptoms of deterioration of social relationships. The length of service was not reflected in the study group in changes in professional effectiveness, and in the case of disillusionment, the highest level of disillusionment was recognized not in this class, but in women with the shortest length of service. In conclusion, it can be said that the length of service as ECD was a variable that significantly co-occurred with changes in the area of the psychophysical strength of the women studied and in the deterioration of their social relationships. Contemporary models of burnout explain its causes by an imbalance in the area of work demands and resources. As a result, psychological tension (an emotional response to exhaustion and anxiety) increases, the level of which is regulated by defensive coping methods activated by employees. They may take the form of the distancing of ECD workers’ in regards to their relations with service recipients [32]. The signs of such relationships can be seen in the results of our study. Higher levels of psychophysical exhaustion co-occurred with higher scores in the deterioration of social relationships, but to a different extent in each class. In Class 3 (emotionally exhausted and distanced with a sense of mission, 4 years of service) high levels of exhaustion co-occurred with high levels of relations’ deterioration, whereas in Class 1 (disillusioned with the risk of changing career, 5 months of service) relations’ deterioration had a lower value than the elevated, above-average level of emotional exhaustion. It was as if the deterioration process had not occurred yet. Additionally, in the other classes where the level of exhaustion had the average value, the level of deterioration was lower than it. The mismatch between demands and resources may concern the exchange: effort put into work–fair payment, as well as the fact that the expectations of employees are not taken into account. This gives rise to a sense of injustice in employees, increasing dissatisfaction, and which has a negative impact on relations with customers [32].

Elevated levels of exhaustion and high levels of disillusionment among women with the shortest length of service may have a number of explanations. One of these is the theory of a deficit in personal resources aimed at protecting the young worker from the “reality shock” that occurs upon starting work [12]. It is also believed that employees at the beginning of their career may experience mismatched job expectations and unfamiliarity with the workplace, have unrealistic expectations about their job, and have no effective strategies to cope with stress, which increases the risk of burnout. A meta-analysis showed a significant negative correlation between the age of employees and PE [41]. This is particularly true for occupations where starting work is not preceded by many years of education and training, during which the future employee gets to know the advantages and disadvantages of their job well and has a chance to develop constructive ways of coping with stress while taking exams. Further reasons include poor introduction and the process of socialization of young workers in their new workplace as well as the measurement error of the healthy worker effect and other non-gender-related changes [12].

It was not possible to differentiate classes due to the complexity of the social roles performed (presumed incidence of WHC conflict in married women and mothers). Neither living in partnerships nor having children, which are potential sources of WHC conflict, turned out to be the factors that would statistically significantly differentiate the results to form independent classes. We observed a higher BMI in unmarried women and a prevalence of unmarried women over women living in relationships in the class with a high level of exhaustion. In view of the fact that these were also the women with the shortest lengths of service as ECDs and that the highest levels of exhaustion were observed in the group with roughly equal proportions of single and coupled women and those with children and without children (Class 3), the explanation of exhaustion risk caused by role complexity was not confirmed in the study. The women with the highest number of children (Class 2) did not obtain more negative results in terms of all burnout symptoms than the other members of the study group. However, at the same time, women with children reported significantly higher levels of emotional exhaustion and had a higher BMI. In summary, the study revealed complex relationships: being a single woman may co-occur with obesity to the same extent as being a mother, whereas being a single woman and being a mother may lead to emotional exhaustion.

The study partially confirmed the assumption of Hypothesis 3. We managed to detect latent correlations between overweightness and obesity and the length of service as ECD, marital status, having children, and occupational burnout, although in the case of the latter variables it was only a tendency. There are research reports in the literature indicating that emotional exhaustion can lead to both a loss of appetite and overweightness due to changes in eating habits and “emotional eating.” These relationships did not appear in our study. The most exhausted women (Class 3) had normal body weight. A normal body weight was also observed in women with the lowest emotional exhaustion (Class 4). In the case of overweightness and obesity, the longest length of service as ECD (Class 2) and presumed WFC appeared to be explanatory factors. Although marital status and the number of children at home did not differentiate between classes, the class with the longest length of service and overweightness (Class 2) had the highest number of women who were in relationships and raising children of all women examined. The group with the second longest length of service and normal body mass index (Class 4) was characterized by a medium severity of burnout symptoms and the highest number of single and childless women. Bearing in mind the previously discussed complex relationships between marital status, motherhood, BMI, and the level of emotional exhaustion, it can be concluded that social role conflict along with the significant length of service as an ECD may mediate between occupational burnout and overweightness and obesity, and the same can be said about being a single woman. However, confirming this assumption requires a different research plan.

In the case of men, obesity was explained by sedentary work and limited physical activity [22]. In Poland, the labor market activation program is addressed to both childless women and those with one and two children. Due to the lack of sufficient supportive measures for working mothers, this increases the risk of stress and burnout and, consequently, somatic diseases. This fact can be used to explain the results obtained in the study.

The application of the Person-Oriented approach and the LPA method allowed us to differentiate the study group and present the results, which are not available in the Variable-Oriented approach. Due to the low rates of burnout in the dimension of professional inefficacy, none of the classes was diagnosed with a full-blown burnout syndrome. The class with the highest indices of psychophysical burnout and deterioration of social relationships reported the lowest intensity of disillusionment, loss of work satisfaction, and a mission to help, as well as professional inefficacy, among all groups of women. They are probably women who are trying to combine work with domestic responsibilities, deriving satisfaction from this fact and trying to maintain a sense of efficacy, probably in both areas. Although their body weight is normal, these women can be identified as a group at high risk of future health problems. The women who developed health problems such as overweightness and obesity had the longest length of service as an ECD with the weekly duty time exceeding 40 h; they were mothers, raising children together with their partners or on their own. The women with a very short length of service who were at an early stage of their career reported an increased risk of psychophysical exhaustion and they were the most disillusioned with their work, which in their opinion, they were doing effectively. Since they were mostly single and childless women, the reasons for the risk of burnout may be sought in both their occupational expectations and workplace organization. The study also discovered a class of women adapted to their occupation, without signs of eating disorders, and with average rates of burnout symptoms.

The study examined the variable of weekly working hours. There are scientific publications which confirm relationships between long working hours, occupational burnout, and the incidence of somatic diseases [42]. The critical point may be reached when the 40 h working week is exceeded [43]. In our study, this point was exceeded in the case of most, or even all, examined women. The number of working hours remains in a linear relationship with occupational burnout in women if variables such as age, marital status, parental status, and household responsibilities are controlled [44]. Even voluntarily increased working hours may co-occur with health problems as a consequence of burnout.

The pandemic seems to have had an impact on the psychological wellbeing of healthcare workers worldwide [45], probably also on ECDs. Scientific research shows that women who work in healthcare faced an increased workload due to increased number of patients with COVID-19 [46]. Women burnout levels among healthcare workers in Portugal (during pandemic) were over four points higher on average comparison with men [47]. High level of anxiety and depression were more common among women–healthcare workers from Wuhan in China [48]. It should be assumed that results obtained by us are influenced by the pandemic crisis.

## 5. Conclusions

The application of a profile approach in setting burnout prevention goals for working women may help to identify a course of action aimed at preventing burnout and burnout-related diseases. The study identified three major classes of women: one at high risk of burnout, another one at high risk of eating disorders, and the third one at risk of existential crisis. The first group consists of women at the beginning of their career, who usually start work just after graduation, even before starting a family. This group requires both careful selection at the recruitment stage in order to choose candidates with a system of values that can be maintained during their professional work and the appropriate preparation of already-selected women for the realities of the profession. This is particularly important in such emotionally charged professions as ECD. It is also vital to begin immediate training in the use of adaptive coping methods to deal with the demands of the job such as callers’ aggression, or dramatic sounds and noises that pose for ECDs the risk of secondary traumatization. The second group is made up of working women who try to reconcile their work with their family roles of mothers and wives. Here, work organization may play a special role, especially in the form of flexible working hours and social and organizational policies supporting the needs of working mothers. It is also important to take actions aimed at changing the expectations regarding the roles performed by a woman, so that work and family life are treated as equal life goals. It is necessary to reduce the complexity of these roles by transferring some of the responsibilities to partners or institutions. Occupational health doctors should research this area and offer working women sufficient psychological support so as to prevent them from using methods of coping with stress that involve health risks such as emotional eating or the unrestricted use of stimulants and drugs. There is also a group of women called an adapted one, without symptoms of the risk of burnout, whose scores in all dimensions had the average value. This was a group including single and childless women, focused mainly on their work. Although clearly visible indicators of risk have not appeared, the problem may concern existential questions regarding both work and lifestyle. The emerging disillusionment and risk of the loss of job satisfaction may be a matter of concern. Due to the relatively long length of service as ECDs of the aforementioned women, actions preventing the loss of their commitment to work may refer to the organization of the workplace in terms of increasing the autonomy of decision-making, providing opportunities for further professional development and promotion.

The study described above, similar to most studies on burnout, was a cross-sectional study, which did not allow us to refer to the alleged causality of the analyzed relationships. This is a significant limitation of this research. In order to show directional relationships, further randomized and controlled experiments with repeated measurement over a period of time should be planned. Only such studies can detect directional changes involving predictors of burnout and its health consequences. We did not have the data from all PSAPs in Poland at our disposal, which means that the results cannot be representative for the entire ECD population. The use of self-report questionnaires (rather than objective behavioral indicators) carries the risk of a number of measurement errors, such as reporting error, method variance error, obtaining data from only one source, and reverse causality error.

## Figures and Tables

**Table 1:** Characteristics of the variables applied in the study. N = 296.

**Parameter**	**M ± SD**
Length of service as ECD	4.3 ± 2.6
PE	21.1 ± 3.6
RD	20.2 ± 4.0
PI	12.3 ± 4.1
DI	18.5 ± 3.4
BMI	23.8 ± 4.2
**Parameter**	**%**
Married	69.5
Unmarried	30.5
Number of children at home	
0	60.6
1	19.0
2	16.5
3	3.2
4	0.3
5	0.3

Note: Variables were expressed as: M = mean ± SD (standard deviation), PE—psychophysical exhaustion, RD—relation deterioration, PI—professional inefficacy, DI—disappointment; BMI—body mass index.

**Table 2:** Comparing model fit for different classes profiles in studied models.

Classes	AIC	BICa	BLMRT (*p*)	Entropy	Latent Class Prob. [Range]	Class Prevalence [Range]
Unconstrained modelss						
1	9315.57	9329.59	---	---	---	---
2	9260.06	9288.61	*p* = 0.0099	0.575	[0.8–0.92]	[0.39–0.61]
3	9239.02	9282.10	*p* = 0.0396	0.761	[0.87–0.92]	[0.18–0.48]
4	9197.48	9255.10	*p* = 0.0099	0.835	[0.86–0.99]	[0.08–0.54]
5	9201.40	9273.55	*p* = 0.2871	0.855	[0.89–0.94]	[0.07–0.41]
Constrained modelss						
1	9315.57	9329.59	---	---	---	---
2	9304.84	9322.48	*p* = 0.0099	0.686	[0.91–0.91]	[0.49–0.51]
3	9278.02	9299.31	*p* = 0.0099	0.795	[0.9–0.96]	[0.02–0.5]
4	9268.63	9293.55	*p* = 0.0198	0.81	[0.75–0.96]	[0.02–0.48]
5	9269.58	9298.13	*p* = 0.3069	0.754	[0.71–0.88]	[0.01–0.44]

Legend: AIC—Akaike Information Criterion, BIC—sample size adjusted Bayesian Information Criterion, BLRT(p)—bootstrapped Lo-Mendell-Rubin test. Lower AIC and BICa values indicate better fitting models. Significant *p*-values for the BLRT(p) indicate that the model is a better fit than a model which is 1 class lower. Entropy is a measure of how accurate a model is at classifying people into latent profiles. This is a number ranging from 0 to 1 and the value of 1 indicates the highest confidence. Latent class probabilities [range] provide a range (i.e., minimum and maximum) of probabilities of assigning each observation to a particular class. The higher the lower value of the range, the greater the likelihood of assigning observations to particular classes. Class prevalence [range]—the percentage of observations in the smallest and largest class. It should be higher than 0.05. In our case, the smallest class in the unconstrained models contains 8% of the observations for the whole group, so it is acceptable.

**Table 3:** Descriptive statistics of variables classes and significance differences between variables by classes. N = 296.

Parameter		Classes				*p*
		Class 1 (N = 38)	Class 2 (N = 161)	Class 3 (N = 24)	Class 4 (N = 73)	
BMI	mean ± SD	22 ± 1.85	25.6 ± 4.93	23.2 ± 3.32	20.8 ± 1.68	*p* < 0.001 *
	median	21.85	25	22.9	20.55	
	quartiles	20.62–22.5	22.09–28.52	20.75–25.13	19.59–22.27	C2 > C3,C1 > C4
The length of service as ECD	mean ± SD	0.69 ± 0.41	5.36 ± 2.15	2.59 ± 1.89	4.37 ± 2.06	*p* < 0.001 *
	median	0.55	5	2.25	4	
	quartiles	0.3–1	4–7	1–3.62	3–6	C2 > C4 > C3 > C1
PE	mean ± SD	22.89 ± 4.08	20.29 ± 3.19	25.12 ± 2.29	20.49 ± 3.34	*p* < 0.001 *
	median	23	21	25	21	
	quartiles	21–25.75	18–22	23.75–26.25	19–22	C3 > C1 > C4,C2
RD	mean ± SD	20.42 ± 4.5	20.04 ± 4.07	24.17 ± 3.17	19.16 ± 3.02	*p* < 0.001 *
	median	20	20	24	19	
	quartiles	18–23	18–23	22–26.5	17–21	C3 > C1,C2,C4
PI	mean ± SD	12.55 ± 3.06	12.17 ± 3.31	12.08 ± 2.54	12.79 ± 1.57	*p* = 0.781
	median	12	12	11	12	
	quartiles	11–14	11–13	11.75–12	11–12	
DE	mean ± SD	19.82 ± 2.02	18.29 ± 4.09	17.04 ± 2.79	18.79 ± 2.05	*p* = 0.001 *
	median	20	18	17	19	
	quartiles	18.25–21	15–21	15–19	17–20	C1 > C2,C3 C4 > C3
Marital status	No	27 (71.05%)	83 (51.55%)	13 (54.17%)	45 (61.64%)	*p* = 0.126
	Yes	11 (28.95%)	78 (48.45%)	11 (45.83%)	28 (38.36%)	
Number of children at home	0	30 (78.95%)	90 (55.90%)	14 (58.33%)	46 (63.01%)	*p* = 0.155
	1	3 (7.89%)	38 (23.60%)	5 (20.83%)	12 (16.44%)	
	2	3 (7.89%)	28 (17.39%)	3 (12.50%)	14 (19.18%)	
	3	1 (2.63%)	4 (2.48%)	2 (8.33%)	1 (1.37%)	
	4	1 (2.63%)	0 (0.00%)	0 (0.00%)	0 (0.00%)	
	5	0 (0.00%)	1 (0.62%)	0 (0.00%)	0 (0.00%)	

Legend: PE—psychophysical exhaustion, RD—relation deterioration, PI—professional inefficacy, DI—disappointment, BMI—body mass index. Kruskal–Wallis test + post-hoc analysis (Dunn–Bonferroni test) for quantitative variables, Chi-squared or Fisher’s exact test for qualitative variables; * Statistically significant (*p* < 0.05).

## Data Availability

Data available on request due to restrictions e.g., privacy or ethical. The data presented in this study are available on request from the corresponding author. The data are not publicly available due to the privacy and professional specificity of the people who took part in this research and who work the Polish emergency system.
